# Biobanking in Israel 2016–17; expressed perceptions versus real life enrollment

**DOI:** 10.1186/s12910-017-0223-8

**Published:** 2017-11-17

**Authors:** Gideon Koren, Daniella Beller, Daphna Laifenfeld, Iris Grossman, Varda Shalev

**Affiliations:** 1 Research Institute, Maccabi Health Services, Tel Aviv, Israel; 2 Tel Aviv University, Tel Aviv, Israel; 3 Teva Inc, Petach Tikva, Israel

**Keywords:** Biobanking, Population based biobanking, Informed consent form, Volunteers, Risk perception

## Abstract

**Background:**

As part of the preparations to establish a population-based biobank in a large Israeli health organization, we aimed to investigate through focus groups the knowledge, perceptions and attitudes of insured Israelis, toward biobanking, and then, after input from focus groups’ participants, to empirically assess the impact of a revised recruitment process on recruitment rates.

**Methods:**

1. Six Focus group discussions were conducted (*n* = 10 per group) with individuals who had routine blood laboratory tests taken in the last 2 years.
2. After addressing the issues raised in the focus groups and revising the recruitment process, individuals undergoing routine blood tests in phlebotomy clinics (*N* = 10,262) were invited to participate in the future biobank.

**Results:**

At the outset of the focus groups there was an overall positive response to the prospect of a population-based biobank. Concerns revolved around infringement on privacy, fears of the “big brother”(e.g. insurance companies), and anxiety about inequality.

Reaction to the language of the informed consent document revolved around concerns over ability to maintain anonymity, to withdraw consent, involvement of commercial entities, and the general tenor of the informed consent, which was perceived as legalistic and unilateral. In general, the longer participants were exposed to discussion about the biobank, the less likely they were to consent to sign in. Overall, only 20% (12) of the 60 participants stated they would agree to sign in by the end of the 2 hour group session.

The feedback obtained from the focus groups was used in the second stage (“real life”) of the study. A team of recruiters received extensive training to enable fruitful discussion and a detailed explanation to questions and concerns raised during the recruitment process. During the second stage of the study, after revising the consent form and training recruiters, a 53% consent rate was observed among 10,262 participants, more than 4 fold higher than estimated at the focus group stage.

**Conclusions:**

The qualitative focus group research helped identify important perceptions and concerns, which were subsequently addressed in the revised consent form and in the discussion the recruiters had with potential biobank donors.

**Electronic supplementary material:**

The online version of this article (10.1186/s12910-017-0223-8) contains supplementary material, which is available to authorized users.

## Background

The scientific community increasingly recognizes the value of biobanking in progressing scientific research into disease and development of novel therapeutic approaches by linking collections of different types of genomic and biological markers in diverse human tissues with personal health information [1–6]. The awareness and attitudes of the general public toward biobanking are critical elements in its success, as the ability to recruit large numbers of subjects define the overall utility of such efforts. While the scientific merit is unquestionable, public concerns have been raised regarding privacy, the potential for discriminating individuals based on genetic and other information generated through the biobank, as well as data ownership and transparency, to mention a few [2, 3]. Accordingly, there is a growing appreciation among members of the medical and academic communities, regulators, and the public at large, that biobanking and associated data collection must not only adhere to tight ethical standards, but also address the needs and concerns of the public participating in it.

The experience learnt through both successful relationships (e.g. Britain’s UK BioBank) where public issues and concerns have been *continuously addressed* in a manner that allowed harmonious function, and adversarial relationships (e.g. Iceland’s DeCode) where there was major criticism for partial non compliance with the issue of individual consent form (1) has led to more attempts to capture the perceptions of participants and address them in a timely and interactive manner. Over the last decade a growing number of studies evaluated public perceptions regarding biobanks in North America, Western Europe, Asia and Australia [2]. The response of different populations varied widely, from strong optimistic support (e.g. Finland and Norway) [4, 5] to suspicion and negative attitudes (e.g. Latvia and Austria) [6]. In most cases, public perceptions were documented through qualitative or quantitative interviews or surveys, touching on core issues such as knowledge, confidentiality, transparency and ownership [2]. In the present study we compared the preparedness of publics to participate, as expressed in the focus groups, with subsequent empirical assessment of real life participation derived from the same population.

According to Israel’s national health insurance law, every Israeli resident is entitled to receive medical treatment at one of four national healthcare providers of their choice, and these providers act as nonprofit organizations. The basket of services each resident receives is determined by law and the funding of these services is transferred to the healthcare providers by the state based on the number of members. Funding comes from the state budget and health tax. Maccabi Health Services (MHS), the second largest health insurer in Israel with over two million members, advanced Electronic Health Records (EHRs), and low drop-out rate, is establishing a population-based biobank platform, associating biological samples with rich retrospective health data and promoting Personalized Medicine.

Population-based biobanks are a useful tool to study complex disease and to characterize genetic variation frequencies, as they collect as many samples as possible from a large donor population in order to achieve a sample size representing the general public. Thus far, a population-based biobank that represents the Israeli population has not been created. MHS has set for itself the target of creating the first population-based biobank representing the Israeli population.

The aims of the present study were twofold:

- To investigate through focus groups the knowledge, perceptions and attitudes of Israelis insured in MHS toward biobanking, and key elements that may affect willingness to participate.
- To examine the rate of successful recruitment of donors already waiting for routine blood draw at Maccabi’s phlebotomy stations, after revising the consent form and training recruiters based on the input gained through the focus groups.

## Methods

The protocol for both stages of the study was approved by Assuta Hospital’s Research Ethics Committee. Consent forms were obtained from participants

1. Focus Groups:

Six focus groups were recruited, 10 participants in each, stratified by age (25–34; 35–49; 50–70 years) and SES (low vs middle-high). Participants were all members of MHS and have had blood drawn for clinical tests within the last 2 years. They had not participated in previous research studies. After Ehtics approval they were randomly selected from a list of Maccabi insured persons who had had a laboratory test in the previous 2 years, according to the selection criteria (age, SES). About one third declined. After an oral consent at the time of the telephone contact, individuals were asked one on one to sign a written informed consent with an option to ask questions.

All groups were conducted by a trained focus group moderator. Participants received a stipend equivalent to $50. All content of the focus groups was digitally recorded and transcribed. After a short introduction, not disclosing any information about biobanking or biological repositories, participants were asked to express their personal understanding of the terms “biological sample repository”, “personalized medicine” and “a social project”. This was done orally, aided by use of drawing and intuitive writing. Participants were then introduced to the concepts and purposes of creating a bio- repository. They were informed that the project has been approved ethically, and that full confidentiality and anonymity of participants and their samples will be observed. They were also informed that, for the purpose of the biobank research activity, each sample will be linked to the electronic medical chart of the participant, and for use of that data (after being de-identified) for research; each study project based on the samples will need to be approved by the ethics review committee separately. Subsequently they were divided into 3 subgroups to discuss and suggest how they would plan such a biobank, what would be the advantages and challenges of developing it, and to list 5 reasons why would someone agree and 5 reasons why someone would disagree to participate.

Subsequently, the participants received the proposed informed consent (Additional file 1) and discussed their perceptions, feelings and attitudes towards it. The form was presented and participants were encouraged to ask questions and make their points of criticism, concerns, as well as suggestions for changes.

A qualitative, descriptive research method as described by Morgan [7] was undertaken to capture participants’ knowledge, attitudes, beliefs and perceptions regarding issues related to biobanking. All materials were reviewed and subjected to thematic analysis conducted by a clinical psychologist experienced in qualitative research, to identify emerging and recurring themes. This analysis was conducted on the transcribed text as well as on the drawings.

Content analysis was pursued [7–9], including thorough review of an investigator specialized in qualitative research, to identify themes related to the issues of biobanking, strengths and challenges, benefits and rewards for participation, or reasons for not joining the biobank. The initial coding was kept close to the participants’ own words by use of short phrases. Patterns were identified in the data as the phrases were reviewed. This pattern identification involved collapsing of phrases into subcategories that were then condensed into broader categories. This interpretive phase re-contextualized the data, resulting in a description of benefits and barriers experienced by the participants.

2) Real Life Recruitment

Using the improved informed consent form based on the input from the focus groups and adding additional accompanying information to elaborate on the consent form (Additional file 2), we set out to simulate the recruitment process we intend to implement for the full project as a 4 week study between Dec 11th 2016 and Jan 5th 2017 in order to test recruitment rates in a real life setting. For this purpose we trained 12 recruiters through a full day training. During the training day the consent form was reviewed in detail (Additional file 2) and all questions were addressed, in addition to preparing the recruiters for potential questions that would come from the field. They also received guidance regarding legal implications, privacy, and medical confidentiality. After a full day training on the study, the recruiters were allocated among 8 Maccabi locations in the central Tel Aviv area (some alone, some in couples and there was also one trio – depending on the site physical size and daily turnover). The recruitment team approached Maccabi members in the waiting area in line for blood tests to be drawn, explaining the purpose of the biobank study, giving them relevant information to take home and those who were willing, signed a consent form to join the biobank.

The recruiting team also marked consent rates by recording the number of people they approached who refused to sign in. The recruiters were also collecting contact details from people who requested more information prior to signing.

## Results

1. Focus groups

Six focus groups of ten members each were recruited, all members of MHS, who have had a clinical laboratory blood test performed during the previous 2 years.

### Response to specific terms

In order to assess the understanding, knowledge and experiences of the population with terms associated with biobanks, the response to several terms was assessed. Prior to receiving any information about the biobank, most participants perceived the term “personalized medicine” to reflect the relationships between themselves and their physicians:

“….a doctor that really knows you and you do not need to repeat to him/her your medical history”..

“…not the way it is today where my doc deals mostly with his computer”.

Only a few related the term to genetic testing that leads to personalized diagnostic tests or therapies, and these were elicited mostly among participants of medium-high SES:

“……based on your DNA they will be able to give you treatment which will be specific for you”. It was also noted that older participants tended to understand the term “personalized medicine” more correctly.

The term “Social Project” was specifically assessed as we wanted to determine whether it increases the level of responsiveness. It was largely perceived as a common effort of donating and giving, without an expectation to receive something in return. It was widely perceived as something that originates by voluntary organizations and not by governments or official institutions. “Social Projects” were associated with values such as love, caring, wish to change, equality, belonging:

‘“being part of a group”,

“making changes for the good”,,

“giving a hand”.

There were no apparent differences among groups, except that among older people (50-70 yr) there were more associations to concrete donations, such as food banks and blood donation. A common “must” for all groups was the noncommercial nature of such projects, with a need to ensure that it is not misused. Participants gave examples of projects that started with these principles; where later it became apparent that “someone was making money”. Similarly, there were views that sometimes commercial entities, such as pharmaceutical companies, wrongly adopt the term “social project” to describe some of their activities. These individuals did not believe that commercial entities should use this term, even if they do something which looks like a social project.

The most common visual associations with “biological sample repository” were test-tubes, generally containing blood. The repository was mostly perceived as a passive collection of samples, with vague ideas as to what it would be used for. A few participants associated the term with DNA, genetics and bone marrow. For the most part, participants did not associate it with science, scientific breakthrough, or exploring diseases. Of interest, there were common negative associative connotations with the repository:

“important to be careful”, “exposure”, “loss of privacy”, “stealing”, “misuse”. It was apparent that most participants were unfamiliar with the objectives of biobanks and the ways scientists are using them.

### Pros and cons for participation

After being introduced to the concepts of biobanks, their objectives and potential to advance medicine, all subgroups expressed ambivalence, with considerations of advancing medicine and improving the quality of life on the one hand, with feelings of danger and misuse.

The most common thematic arguments in favor of participation in the bio repository included the following:

- Improving the quality of life through diagnosing, combating and preventing diseases, and by developing new medications. In most cases, these statements were general and did not correspond to specific diseases or conditions.
- Helping the next generations, often by the hope of preventing diseases to pass to next generations. This appears to be one of the strongest arguments in favor of biobanking:
  “ I love the idea that my donation, which will not help me, will help our children”; “it will take many years to see benefits, but the children will benefit from it”.
- Helping other people, with the wish to be part of a big effort to do good, and the expectation to be acknowledged for it. In that context, some of the participants wished to receive updates of the research and discoveries achieved with their donations
  “……so you know that something is done with it and the blood does not just sit there in a fridge”.
  The challenges and objections to the biobank were elicited without apparent age differences, although older participants (50-70y) appeared to raise somewhat less negative arguments than younger people. The most common themes were as follows:
- Concerns of breaching privacy and medical confidentiality. This could be by illegally entering the biobank, and by misuse of the data.
  “…..I personally would not participate. I do not wish my medical information to be out there. You never know where it can end up”.

“I will need to know in detail what they are doing to protect my information”.

- Fears of “big brother”, such as insurance companies, potential employers etc. Part of the respondents felt that people with known diseases will not participate so that their data will not be available to hackers. Others felt that even genetic information may bear similar risks
  (“…….insurance companies will not agree to insure people with certain genes”.
- Concerns about commercialization. Virtually all participants expressed objection to the fact that their tissue donations will be used by commercial entities to make money. (“even if there is oversight- the fact that commercial interests are involved- I do not trust the results”).
- A concern was raised mostly by people in the low SES strata, that even if the biobank leads to medical improvements, such progress will benefit mostly the rich, despite everyone contributing equally.
- Fears of becoming aware of incidental actionable genetic findings were raised by a minority of responders, though most participants found this to be an advantage to participants.

### The consent form (Additional file 1)

Participants raised numerous issues regarding the consent form. Some wondered whether their samples can really remain anonymous in view of linking it with their de-identified medical records:

“….it is not really anonymous if someone can link my sample to my medical file”.

Participants took issue with the lack of pre-specified time limit on the use of their samples, including subsequent to the subject’s death:

“In every other agreement there is an expiration date”. Some doubted whether in cases where they opt out after donating a sample, the existing samples will really be discarded and unused. Many disliked the sentence asking to use left-over samples collected from the individual in the past.

Allowing commercial bodies to be part of the research effort induced a negative reaction in most responders, as mentioned earlier.

Several participants objected to the statement that they will not receive anything in return despite commercial entities being potentially involved:

“I do not mean money. It can be a privilege within the HMO”. “What’s in it for me”. Among possible payback methods, participants mentioned receiving a genetic interpretation, a warning of a medical problem that was identified, priority in receiving novel therapies or drugs that were invented from the research and discounts in paying their health insurance.

The one sided tenor of the consent form was a genuine hurdle for many, feeling that it was written in an unfriendly legalistic manner, protecting the biobank and the HMO planning it and not the volunteering participants.

### Group dynamics

As a general trend, during the focus group discussions and interactions, the longer the participants discussed the biobank amongst themselves, its processes and challenges- the less likely they were to agree to sign in. The observations indicate that the longer the discussion, more doubts and fears were voiced which remained unbalanced by lack of answers by other participants, as, by design, the groups’ mentors did not intervene with any input. Overall, at the end of the focus groups only 20% [10] of the 60 participants in the six groups would agree to sign and participate.

2) Real Life Recruitment

**Fig. 1:**
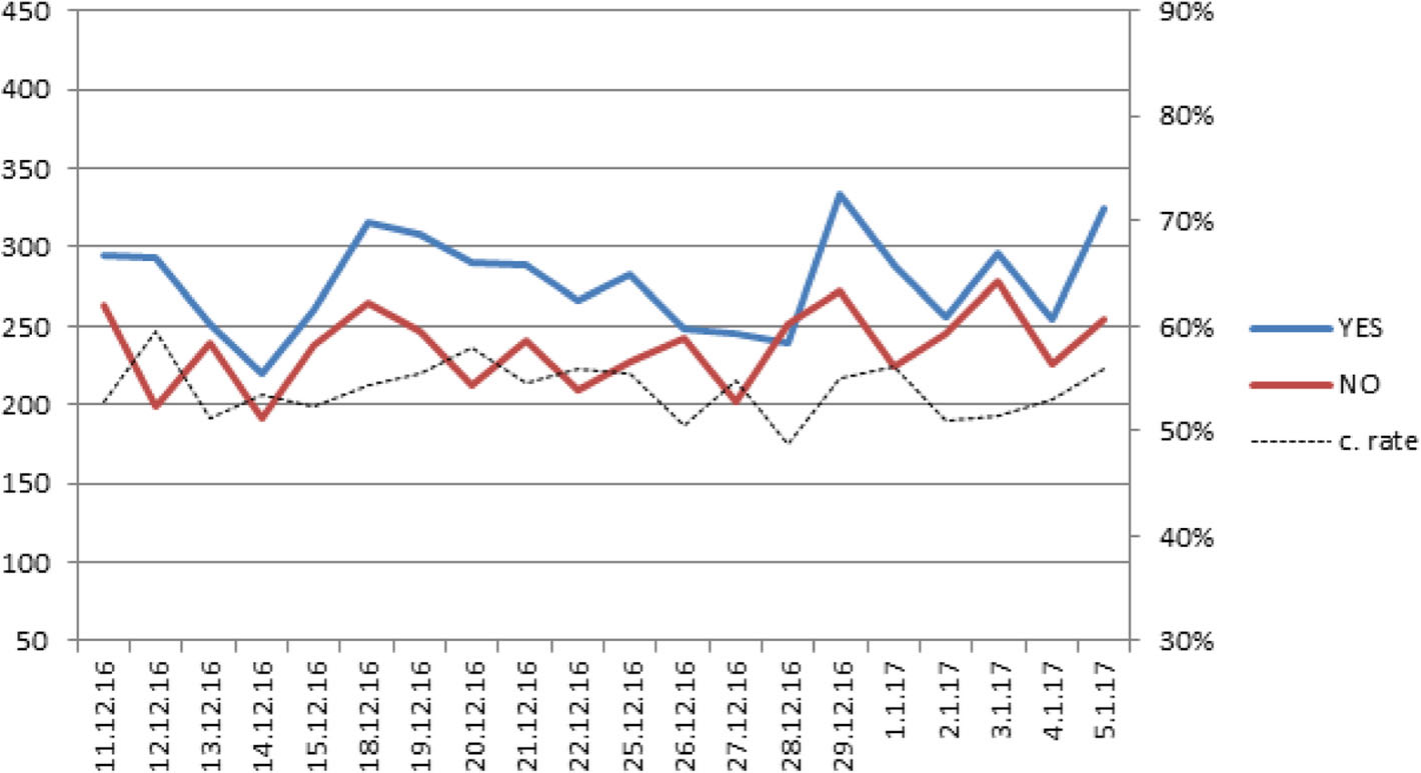
Rates of recruitment and refusal in the real life experiment

Ten thousand, two hundred and sixty-two individuals attending MHS laboratories for routine clinical blood tests were approached and invited to participate in the biobank by signing for donation of their samples. Of these, 5542 (53%) agreed and signed the improved consent form after explanations by the recruiters (38% males 62% females) (Fig. 1). Seven people who had originally signed a consent form later re-contacted us to withdraw their consent. On average, it took 10–15 min for candidates to read the consent form and another 5–15 min to address all queries by subjects before signing the consent form, although some took the written brochures to read at home before they sign.

## Discussion

The present study describes the development of an informed consent form for a population-based biobank based on direct feedback from the public through focus groups which addressed their concerns and fears. At the outset of the focus groups, it was apparent that participants’ familiarity with the concepts of “personalized medicine” and “bio repository” was limited. This reality stressed the need to ensure that these concepts are reflected in the revised consent form. The exploration of these terms allowed the researchers to comprehend what lay people are likely to know, or not, and, in this way, what is missing and should be stressed. The focus group discussions offered critique by participants, which was brought back and discussed by our team, leading to changes in the consent form. For example, it is evident that the revised consent form is much morefriendly, and does not “legalistic”, as a contract typically prepared by lawyers. This was based on critique by members of the focus groups.

Similar to other studies in the field of biobanking, the Israeli participants defined succinctly their concerns regarding potential breach of privacy, confidentiality, and misuse of the data by “big brothers” [1–3]. Of importance, it became apparent that individuals who appeared relatively enthusiastic to join at the outset of the focus group interactions became more reserved as they were exposed to fellow participants’ concerns without receiving, by design, more informative answers from the group’s moderator. In contrast, during the second stage of the study, in which real life recruitment was assessed, experienced recruiters trained by us were available to address many of the concerns elicited in the focus groups, probably contributing to the high recruitment rates. This is in line with other experiences elsewhere, where with increasing knowledge came more willingness to join (2).

As shown by other groups [2] it was apparent that there is a need to ensure that the consent to participate in biobank donation is optimally informed. It has been also shown that the formal consent form needs to be supplemented by plain language materials [11] and an opportunity for participants to ask questions and receive further clarifications on a variety of concerns and topics [9]. We achieved this goal by preparing an expanded document that was given to participant by the recruiters, allowing a more complete communication with potential volunteers, addressing a wide range of questions (Additional file 2).

A major issue in obtaining an informed consent is the ability of lay people to comprehend the information conveyed, both in terms of its volume, as well as the language and terminology used. In our case, respondents of the focus groups felt that the language was unilateral, contract like, focusing on the interests of the bio repository more than on the interests and the rights of the participants. Following the overall negative responses of the focus groups’ participants, where only 12 out of 60 would agree to sign the informed consent as presented, we were encouraged by the over 53% agreement to join and donate to the biobank in the real life recruitment. This can be partially explained by the improvements made to the consent form following the focus groups. In addition, the knowledge offered by the trained recruiters enabled them to address and counter concerns (Additional file 2), in contrast to the focus group setting. This is consistent with studies showing the need for communication with individuals after receiving consent from a biobanking project [12] to ensure understanding and improve acceptability.

Of all potential issues and challenges raised by our participants, the strongest negative responses were associated with the prospect of commercialization of the biobank with the feeling that a private enterprise is profiting from samples given voluntarily and with no conditions. The establishment and maintenance of a bio repository is expensive and the public responding negatively to the commercial aspect is often not aware of this fiscal reality. One of the few studies that have tackled the commercialization issues in details came from Australia. Many participants who had a prejudice against commercial entities potentially profiting from public tissue donations, agreed that a biobank with commercial involvement is a better choice than not having a biobank at all [10]. One important strategy to address such concerns is through transparency - tight governance of the biobank and oversight by a research ethics committee that sanctions and approves every research project, need to be made known to potential participants in an effort to increase public trust [10]. In order to address this issue as well within our own setting a paragraph was dedicated to this subject within the accompanying information to the revised consent form (Additional file 2: “Creation and research activity”) in which we detail MHS as well as the biobank being not-for-profit and having all funds gained returned for the continued existence of the biobank for future research in order to achieve the goal of advancing medicine.

Incorporating the response of the various focus groups has led to the development of the supplementary data that accompany the consent form (Additional file 2), as well as changing some of the language of the consent form itself. As importantly, these changes were used to inform the biobank recruiters during the “real world” recruitment. It appears that in real life, potential participants are asking questions that reflect the content of what was alluded to during the focus groups and that on average these questions take between 5 and 15 min to address. Participants not only received an “on the spot” reply but also received a hard copy of this detailed information and contact details to take home with them, which increased confidence in the commitment of the biobank towards them.

These results reflect the response to a population-based biobank, and may differ in specific disease-based repositories, where potential participants are patients with the specific conditions and their family members, who may be much more familiar with the databank aims and rationale.

## Conclusions

In summary, the utilization of focus groups prior to establishing specific recruitment processes for a biobank appears to have worked well in our environment. The qualitative focus group research helped identify important perceptions and concerns of participants, which were subsequently addressed in the revised consent form and in the discussion the recruiters had with potential biobank donors. The impact of these changes is reflected in the over 4 fold increase in consenting individuals from rates predicted by the focus group. Our study suggests that conducting focus groups prior to the tremendous efforts of to establishing a biobank, may increase the utility and effectiveness of the final aim.

## Additional files

Additional file 1: Original consent form, as presented to focus group participants. (DOCX 17 kb)

Additional file 2: Updated consent form and additional information as used in real-life recruitment study. (DOCX 31 kb)

## Abbreviations

HMO: Health Maintenance Organization
MHS: Maccabi Health Servises
SES: Socioeconomic status
UK: United Kingdom
