# Supplementary material for: Biobanking in Israel 2016–17; expressed perceptions versus real life enrollment
Source: BMC Med Ethics. 2017 Nov 17;18:63. doi: 10.1186/s12910-017-0223-8 (PMC5693555; doi:10.1186/s12910-017-0223-8)
Supplement: Supplementary file 1 — Original consent form, as presented to focus group participants. (DOCX 17 kb) [file 12910_2017_223_MOESM1_ESM.docx]

Biobanking: Additional file 1

**Additional file 1: Original consent form, as presented to focus group participants**

**SIGNATURE FOR VOLUNTARY CONSENT/AUTHORIZATION**

**Maccabi Healthcare Services - Center for Precision Medicine Research – MaccabiTech**

Project Title: Collection and storage of biological samples and data in the biological sample repository of Maccabi Healthcare Services (“Maccabi”) to be used in current and future medical research, including genetic research.

I hereby confirm that I have received information regarding the biological sample repository of Maccabi (“Maccabi bio-repository”), I have read and understood the accompanying information (or it has been read to me), and I have had the opportunity to ask questions about it and my questions have been answered to my satisfaction. By signing this Informed Consent Form I consent voluntarily to have my samples stored in the manner and for the purpose indicated in the information sheet: to donate my biological samples (blood, saliva, urine, feces, or any other biological sample) for storage in the Maccabi bio-repository for the purpose of conducting research projects which all have been duly certified. I understand that the Maccabi bio-repository will enable research projects such as identifying risk factors for the development of diseases or predicting response to various kinds of treatments. I understand that the purpose of these research projects is to advance knowledge in biological and medical sciences and to improve the diagnosis, treatment and overall quality of life of persons suffering from different diseases.

I understand that information from my electronic medical record will be used in these research projects in the same manner and for the same purpose as indicated above for biological samples. In addition, I consent voluntarily to the use of existing biological samples which have been sampled from me in the past, prior to my signing this consent form, to be used for research purposes, if such samples exist.

I understand that any and all information about me which will be used for research will be de-identified (e.g. coded and without my identifying information), and only Maccabi will be able to link my identity to my samples or medical information.

I understand that my consent is not limited in time, unless I choose to withdraw from the bio-repository, and my consent will continue to be valid after my death. I understand that it is my right to withdraw from the bio-repository at any time and I can do so according to the withdrawal process described in the accompanying explanatory information. I understand that my decision to participate or withdraw from the bio-repository will have no effect whatsoever on the decisions relating to the medical treatment that I or members of my family will receive.

I declare and confirm that it has been explained to me that Maccabi is establishing the bio-repository and intends to initiate collaborative research with external partners, including academic institutes and commercial entities, for the purpose of advancing scientific and medical knowledge by use of the bio-repository.

It has been explained to me and I understand that not all of the donated samples will necessarily be used in any research project. It has also been explained to me that among the samples which will be used for research and will undergo genetic sequencing, not all samples will necessarily be tested for all known genetic variances. I additionally confirm and agree that signing this consent form does not give me a right to demand that Maccabi perform any given test on my biological sample, and any decision regarding tests to be performed on my samples will be Maccabi’s alone.

By signing this form I consent to Maccabi’s right to contact me directly to request additional biological samples to be used for the same goals and purposes for which I am consenting to donate my samples now and under the same terms.

Genetic findings

I know and understand that genetic variations found incidentally are an inevitable consequence of genetic testing and may be discovered while performing research on the samples of the Maccabi bio-repository. I confirm that it has been explained to me that I may be contacted in the future regarding Information about my increased risk for a health problem. I understand that only a small fraction of the participants of the bio-repository are expected to be re-contacted with such information and that I am not to assume any conclusions if I am not re-contacted (for instance I cannot assume my sample has not been genetically sequenced, nor should I assume that my sample has been genetically sequenced and I have been found to be variant free).

I confirm and agree that my participation in the bio-repository is voluntary and that I will not be receiving any or all financial or proprietary rights from my participation, nor am I entitled to any financial gain which may be a result of research done by the bio-repository. I hereby wave my rights to my biological samples and the information created from them, including rights to any commercial outcome. Given all of the above, I understand that my participation in the Maccabi bio-repository project is a substantial contribution to promoting scientific and medical knowledge, with the aim of improving the diagnosis and treatment of people suffering from various diseases, and for the advancement of medicine in Israel and around the world.

Name:________________ Signature: _______________
ID number:_____________ Date: ___________________

Declaration of obtained consent:

Consent was obtained from the above-named donor after a thorough explanation of all of the above and after verification that he or she has read both the Consent Form above and the additional Information Sheet below, and after ensuring that he or she understood my explanations.

Name:____________ Signature:______________
