# Supplementary material for: Biobanking in Israel 2016–17; expressed perceptions versus real life enrollment
Source: BMC Med Ethics. 2017 Nov 17;18:63. doi: 10.1186/s12910-017-0223-8 (PMC5693555; doi:10.1186/s12910-017-0223-8)
Supplement: Supplementary file 2 — Updated consent form and additional information as used in real-life recruitment study. (DOCX 31 kb) [file 12910_2017_223_MOESM2_ESM.docx]

Biobanking Additional file 2

**Additional file 2: updated consent form a d additional information as used in real-life recruitment study**

Hello, and thank you for joining “Tipa”("a drop") bio-repository. Please read this form and the accompanying information prior to signing. Please ask any question to assure full understanding. Note: this for m shortly outlines all the topics, therefore it is essential that you read the additional material before giving your consent.

“Tipa”’s objective: Collection and storage of biological samples for use combined with medical data in current and future medical research, including genetic research, in areas such as detection of disease causation or personalized drug treatment. I understand that “Tipa” is subject to regulations such as rules requiring meticulous storage of biological samples and maintaining confidentiality of information. The samples in “Tipa” will be coded, detached from identifying information. When medical data will be needed for research, it will be de-identified to assure anonymity. Only a limited few of the repository staff will have the ability and authority to make the connection between me and my sample if needed.

I, the undersigned, hereby confirm I understand and agree on my own free will:

- To donate samples such as blood, saliva, which are being collected as part of my medical treatment
- To have my samples used in research aimed to advance knowledge in biological and medical sciences in Israel and world-wide, in order to improve the diagnosis and treatment of persons suffering from different diseases
- my samples will be used in studies which may identify my genetic code and link it to my medical record
- all studies using the repository are subject by law to approval of an ethics committee (aka Helsinki committee)
- my consent is not limited in time and will continue to be valid after my death
- I can withdraw from “Tipa” at any time (as detailed in the accompanying document) and my samples will be discarded
- joining or withdrawing from “Tipa” will have no effect on the medical treatment given to me or my family
- studies at “Tipa” will include collaboration with external partners such as academic or commercial institutes
- not all of the donated samples will necessarily be used in any research project or undergo genetic analysis
- Maccabi has no obligation to perform any given test on my donated biological samples
- if, while used for research, information on genetic variance of clinical relevance is discovered in my samples, (as defined by “Tipa” genetic advisory board), I will be contacted by a “Tipa” representative for counseling as detailed in accompanying information. Only a small fraction of participants are expected to be re-contacted
- I understand I should not come to any conclusions if I am not re-contacted
- I understand that the bio-repository is for research purposes only and is not to be perceived as a tool for managing my health
- I hereby waive the rights to my donated samples. My participation is voluntary, for the purpose of advancing medicine. I understand that am not entitled to any financial gain or proprietary rights (including commercial rights) which may result from the findings of any research project or other product derived from them.

Name:________________ Signature: _______________
ID number:_____________ Date: ___________________

Declaration of obtained consent:

Consent was obtained from the above-named donor after a thorough explanation of all of the above and after verification that he or she has read both the Consent Form above and the additional Information Sheet below, and after ensuring that he or she understood my explanations.

Name:____________ Signature:______________ Date:_____________

**“Tipa” bio-repository additional information to Consent Form**

hello,

You have been asked to contribute biological samples of yours to the “Tipa” bio-repository, established by the research institute of Maccabi Healthcare Services (“Maccabi”). Collected samples from all participants will be used to promote scientific, medical and genetic research today and in the future. In this pamphlet you will find detailed information on the repository. It is important you read these pages in order for you to be able to give your consent based on a full understanding of the project and its goals. Don’t hesitate to ask the team member that approached you any questions. If you decide to join “Tipa” fill out your details in the consent form. Please note – at the bottom of the form you will be able to find details regarding the “Tipa” website and contact information.

**What is medical research?**

The aim of any medical research is to broaden and develop the existing knowledge in order to advance medical treatment in the future – improve diagnosis, treatment, disease prevention, and causal factors for disease. The better we understand the causes of disease, and how to treat it, the more we will succeed in reducing the suffering of those with illness.

Medical research can be carried out by giving a new experimental drug to patients. Another way to perform medical research can be by conducting genetic tests. The genes we inherit from our parents do not only determine the color of our eyes or our appearance, they are also connected to our health: they can increase our chances of developing disease, or the opposite – reduce our risk or protect us from disease.

Today we know that with the help of DNA testing we can find out for example if someone has an increased risk to develop some types of cancer. Some genetic markers are inherited and shared among family members, while others are unique to specific individuals. Research of genetics investigates groups of people in order to learn more about the connection between their genes and their health. Such connections are not only related to the development of diseases but can also teach us why different individuals have different responses to the same medication – it can cure one person and have no effect on another. The more information we accumulate the better understanding we will have regarding the role of genetics in our health and find a better way to personalize the treatment we give each person based on their genetic profile. This type of medical treatment based on genetic profiling is called “personalized medicine”.

Every medical research study, of any kind, must be first approved by an ethics committee. The Ethics committee (also known in Israel as the “Helsinki committee” or around the world as the IRB – institutional review board), is a committee made of leading physicians and representatives of the public. The committee acts on behalf of the ministry of health and is responsible to examine the ethical aspects of medical research projects. The committee acts independently and is in charge of protecting the rights, safety and well-being of the participant of the study. Each study is submitted for review by the committee and only it has the power to approve the study execution. "Tipa" itself will have to receive a Helsinki approval for each project separately. In the same fashion, the "Tipa" biorepository has received ethical approval from the Ministry of Health's high committee on human subjects research, to store and distribute your de-identified samples and data in future ethically approved research studies.

**What is the connection between the bio-repository and medical research?**

The “Tipa” bio-repository will enable researchers to conduct studies attempting to address questions as detailed above. We are creating this repository in order to facilitate medical research by collecting samples from different people of all ages, of all health backgrounds, which consented to participate. By having these samples available in advance in the repository, many different medical questions can be raised and tested: one study could focus on cancer, another on Parkinson’s disease, and another can study drug resistance etc. through this model, “Tipa” can help improve medical knowledge and contribute to the future application of such knowledge by doctors when treating their patients.

**How will the “Tipa” repository work?**

The “Tipa” bio-repository is a storing repository meaning: It will store various types of biological samples in the recruited conditions to enable their future use in medical research. Samples to be collected for example: blood, saliva, urine feces. Samples will be collected from participants, members of Maccabi, ill or healthy. The samples by themselves are not enough to conduct research. In order for these samples to be used there is a need for up-to-date medical information on the donors. This information is recorded in your electronic medical record at Maccabi.

The samples in “Tipa” will be coded, detached from identifying information. When a research project raises and will want to make use of certain samples from the repository, only then will a connection be made between the samples and their donors information. This connection will be made with the sole purpose of extracting the relevant information of the sample donors, and still then, after extracting the required data, it will be de-identified, removing all identifying details, before being used in research to assure anonymity (more on this detailed in the following chapter regarding “Privacy“).

**Objectives**

The purpose of collecting and storing biological samples is for them to be used in research. As detailed above, in order to conduct research information from electronic medical records is needed as well. The combination of samples and data will enable researchers, from Maccabi or external to Maccabi, to conduct research projects for the advancement of medicine. Every study will be first duly approved by the ethics committee (Helsinki/IRB). Studies of the “Tipa” repository will be in accordance to the following objectives:

1. To identify risk factors for the development of diseases
2. To detect populations at high risk for the development of diseases
3. To identify markers for the early detection of diseases
4. To understand the susceptibility or resistance of cells (in health or disease) to various kinds of treatments
5. To research diseases
6. To conduct any other research approved in the future by the ethics committee

**Participation**

Maccabi invites you to take part in the bio-repository project by donating biological samples which will be collected and stored with samples from other consenting donors. The samples will be collected during visits you make to the Maccabi sample collection stations as part of your regular medical treatment. For example, when you come to the blood-draw station in order to give a sample for a blood test requested by your treating physician, the same needle which has already been inserted in to your arm will be used in order to collect another tube of blood (equivalent to about 3 spoonful’s) which will be sent for storage. All samples collected will be under the responsibility and liability of Maccabi.

**Participation is voluntary**

Your decision whether or not to participate in the “Tipa” is voluntary and you have the right to cancel your participation at any time (as detailed in the “withdrawal section later on). Your participation will have no effect whatsoever on the decisions relating to the medical treatment you will receive. You will not receive any financial or proprietary rights (including commercial rights), nor are you entitled to any financial gain which may result from the findings of any research project or other product derived from them. By consenting you are agreeing to donate your samples in order to advance medical knowledge. The outcomes of the results conducted at “Tipa” may be used in the future to better the lives of people in Israel and around the world.

**Creation and Research activity**

Maccabi is a not-for-profit organization. According to Israel’s national health insurance law, every Israeli resident is entitled to receive medical treatment at one of four national healthcare providers of their choice, and these providers act as nonprofit organizations. The basket of services each resident receives is determined by law and the funding of these services is transferred to the healthcare providers by the state based on the number if members. Funding comes from the state budget and health tax.

As a nonprofit organization Maccabi is responsible for covering its own operational costs. The same goes for the “Tipa” repository: since the establishment of a bio-repository is a complex initiative requiring costly technological and physical infrastructure, the establishment of the repository will be financed by donation received specifically for this purpose (and not from the Maccabi funds). In order to fund the activity of “Tipa” and enable it to continue to function as a research hub, research projects will be conducted as research collaborations. These research collaborations will be with groups which share the goals of “Tipa”: to improve the diagnosis, treatment and overall quality of life of persons suffering from different diseases. Such partners may be external partners, from Israel or abroad, including academic institutes and commercial entities. All funding from these studies will return to “Tipa” to fund it conscious existence for research. By participating in the bio-repository you will provide an important contribution towards achieving these goals for the advancement of medicine in Israel and across the world.

**Privacy**

“Tipa” is bound by the laws applying to the health system in Israel, which include meticulously storing biological samples in a professional manner while strictly maintaining the anonymity of the sample donors and all their medical information. Anonymity of all participants will be guarded as required by law under Israel’s Privacy law and Law for genetic information

Samples will be stored in “Tipa” with a code and without any identification details. As part of any research conducted at “Tipa”, all of your sample of personal details, as well as your medical information or genetic information will remain confidential. Information used for research will be coded and stripped of any identifying details. Such information is called DE-IDENTIFIED information meaning it is coded and without information such as social security number, address, etc. Details of the bio-repository donors will be restricted and only a limited few of the repository staff will have the ability and authority to make the connection between you and your sample if needed.

It is important to re-emphasize that all research studies conducted at “Tipa” require approval by the ethics committee (Helsinki) as detailed above in the chapter on medical research. The ethics committee is made of leading physicians and representatives of the public, who decide whether to approve any and all studies involving human subjects and it is the committee responsibility to make sure studies comply with the ethical boundaries that include protecting the confidentiality and privacy of the participants in the “Tipa” bio-repository.

“Tipa” is committed to applying measures to maintain privacy and security of the samples and all information. The samples will be stored in special freezers with limited access only to the bio-repository staff. The samples will be coded in the computerized systems. The identity of the donors will be kept separately, safely stored, under extensive security systems, in order to verify confidentiality. At the end of any given research project, left-over samples will either be returned to the repository

**You might be re-contacted if genetic findings are discovered in your samples**

As part of the activity of the “Tipa”, a professional genetics advisory board will be established. This advisory board will be in charge of creating a list of genetic variations which should be reported back to the donor of the sample, based on up-to-date international criteria. This list of genetic variances may change from time to time based on the professional opinion of the board members.

It must be emphasized that not all of the donated samples will necessarily be used in any research project, as each study will have different criteria for choosing samples. In fact, only a small fraction of the participants are expected to be re-contacted with any uncovered genetic information. Having said this, no assumption can be concluded if you are not re-contacted, meaning: you cannot conclude if you sample was used in a certain study, nor should you conclude that your sample has been genetically sequenced and you were not contacted since no variance has been found. Not all samples used for research and will undergo genetic sequencing and will not necessarily be tested for all known genetic variances.

What could be discovered in your DNA? You may be found to be at increased risk for developing a certain illness which may be prevented by taking pre-emptive measures. Or for example a certain medical treatment might be found to be unsuitable for you. As mentioned, the list of genetic variants will be prepared by the genetics advisory board based on up-to-date international criteria. If a genetic variation from the list is found in your sample, you will be contacted by a representative of “Tipa” which is a personnel trained for the communication of genetic findings.

It is important to clarify that genetic information which may be reported to you, is important for your health and may also be important to your family members. Maccabi will make available to you all necessary means to help you understand the information you receive. The decision on what actions to take as a result of this information is yours alone. However, we recommend consulting a medical expert (family physician or genetic counselor) who can assist you in making the choices best suited for you.

**Advantages and risks**

Are there advantages to my participation?

As you are contributing samples to be collected and used in future research, the results of which we cannot anticipate, we cannot predict what impact your contribution to “Tipa” will directly have on the medical treatment you will receive in the future. Having said that, the main advantage of your participation is that research projects based on the “Tipa” samples may enable scientists to make the connection between a disease, phenomenon, or trait, and the underlying genes. The hope is that with the help of your sample, and samples donated by others, researchers will be able to develop better tools for diagnosis and treatment and help people in the future.

As detailed above, there is a chance that as a result of your participation you will be notified regarding genetic information which may affect your health, but this is only expected for a small fraction of participants.

Are there risks to my participation?

There is no medical risk associated with your donation of a sample to the bio-repository.

**Duration of “Tipa”**

The “Tipa” bio-repository is a long term project. We would like “Tipa” to enable as many studies as possible to contribute to the medicine of the future. For this reason there is no planned end date for “Tipa” and that is why your consent is requested with no time limitation and to continue also after your demise. Your samples and information will be kept until they are no longer useful for research, or until the “Tipa” project ends. If we will need to close “Tipa”, Maccabi will decide, with the help of its advisory board, how to transfer any remaining biological samples to a research program with similar objectives.

**Withdrawal**

It is your right to withdraw from the “Tipa” at any time. If you decide to withdraw for any reason, you can do this by filling out a withdrawal form which will be forwarded to the repository management through the administration offices at each Maccabi branch, and your samples will be destroyed. Please note that if one of your samples was already sent to be used for research prior to your notification of withdrawal, it will remain in use of this specific research project, while all other remaining samples in the repository will be destroyed and will not be used in any research from the moment your withdrawal is registered.

**Updates and contact details**

We would like to thank you for joining “Tipa” – we see you as our partners in promoting research. Your willingness to donate samples will enable us to create an important research tool which will facilitate medicine advancing research.

We invite you to get involved and stay updated on our activities. Details and updates will be available to you on the Maccabi website: [www.maccabi4u.co.il](http://www.maccabi4u.co.il) . Additionally, once you have joined the project, a special personalized page will open for you on you online services page online.maccabi4u.co.il (and on the Maccabi App). Once you enter the site with your personal password, you will be able to view personalized information regarding your samples.

You may contact us by email at [tipa@mac.org.il](mailto:tipa@mac.org.il) or by phone: 03-7462000
